# A novel fluorescent cardiac imaging system for preclinical intraoperative angiography

**DOI:** 10.1186/s12880-021-00562-y

**Published:** 2021-02-25

**Authors:** Sara Mashalchi, Sara Pahlavan, Marjaneh Hejazi

**Affiliations:** 1 Medical Physics and Biomedical Engineering Department, School of Medicine, Tehran University of Medical Sciences, 1417613151 Tehran, Iran; 2 Department of Stem Cells and Developmental Biology, Cell Science Research Center, Royan Institute for Stem Cell Biology and Technology, ACECR, Banihashem St., Resalat Highway, P.O. Box: 16635-148, 1665659911 Tehran, Iran; 3 Research Center for Molecular and Cellular Imaging, Bio-Optical Imaging Group, Tehran University of Medical Sciences, Tehran, Iran

**Keywords:** Fluorescent cardiac imaging, Indocyanine green, Intraoperative angiography, Rodents

## Abstract

**Background:**

Intraoperative coronary angiography can tremendously reduce early coronary bypass graft failures. Fluorescent cardiac imaging provides an advanced method for intraoperative observation and real-time quantitation of blood flow with high resolution.

**Methods:**

We devised a system comprised of an LED light source, special filters, lenses and a detector for preclinical coronary artery angiography. The optical setup was implemented by using two achromatic doublet lenses, two positive meniscus lenses, a band-pass filter, a pinhole and a CCD sensor. The setup was optimized by Zemax software. Optical design was further challenged to obtain more parallel light beams, less diffusion and higher resolutions to levels as small as arterioles. Ex vivo rat hearts were prepared and coronary arteries were retrogradely perfused by indocyanine green (ICG). Video angiography was employed to assess blood flow and plot time-dependent fluorescence intensity curve (TIC). Quantitation of blood flow was performed by calculating either the gradient of TIC or area under curve. The correlation between blood flow and each calculated parameters was assessed and used to evaluate the quality of flow.

**Results:**

High-resolution images of flow in coronary arteries were obtained as precise as 62 µm vessel diameter, by our custom-made ICG angiography system. The gradient of TIC was 3.4–6.3 s^−1^, while the area under curve indicated 712–1282 s values which ultimately gained correlation coefficients of 0.9938 and 0.9951 with relative blood flow, respectively.

**Conclusion:**

The present ICG angiography system may facilitate evaluation of blood flow in animal studies of myocardial infarction and coronary artery grafts intraoperatively.

## Background

Novel intraoperative coronary angiography technology based on fluorescent cardiac imaging (FCI) aims to resolve the technical short comes of conventional techniques. Among conventional systems, thermal coronary angiography reported to provide low resolution especially in depth, in addition to dependency on expert-based analysis. Moreover, ultrasound transit-time flow measurement (TTFM) shows lack of visualization and quantification, is operator-dependent, and exerts low resolutions over high-frequency epicardial echocardiography [1–3]. Graft failures after coronary artery bypass grafting (CABG) mostly occur due to the quality of the target vessel, insufficient flow in the bypass conduit as a result of inability to compete the native coronary arteries, as well as potential technical failures during surgery [4, 5]. Thus, an intraoperative real-time visualization system with high resolution and automaticity which is user-independent and possesses quantitation properties, would greatly influence the success of surgery. FCI offers a non-invasive, high-resolution and quantitative system for coronary vessel visualization, and intraoperative assessment of bypass grafts and myocardial perfusion [6–8]. However, FCI has not yet replaced conventional techniques due to some technical limitations such as the insufficient penetration depth of laser beam, influence of adjacent tissues on FCI efficacy and lack of suitable quantification [7, 9]. Detter and colleagues could successfully enhance FCI efficacy by combining quantitative assessments of myocardial perfusion and FCI imaging for evaluation of graded coronary stenoses, thus indirectly identifying the bypass graft success [8]. Furthermore, they developed a quantitative system for validation of FCI by differentiating between flow-limiting and non-flow limiting coronary stenosis, to indirectly predict the success rate of bypass surgery [10]. Yamamoto and colleagues introduced blood flow as a measure of coronary bypass stenosis by analyzing indocyanine green (ICG) intensity within the coronary graft [11]. We also developed an FCI system for visualization and assessment of coronary artery blood flow in preclinical models, as a basis for development of intraoperative coronary angiography; our compact design included four-lenses, a band-pass filter and a detector, and resulted in high-resolution imaging of coronary artery flow. Furthermore, our in-house built FCI system employed LED as the light source which replaced the laser pulses used in other systems. The LED light was paralleled using a collimation lens for homogenized lightening of the sample. This FCI could successfully image coronary artery flow in ex vivo rat hearts. Furthermore, a high correlation between relative blood flow and time-dependent fluorescence intensity curve-associated parameters, was obtained.

## Methods

Design, implementation and optimization of FCI.

### Mechanical apparatus

**Fig. 1:**
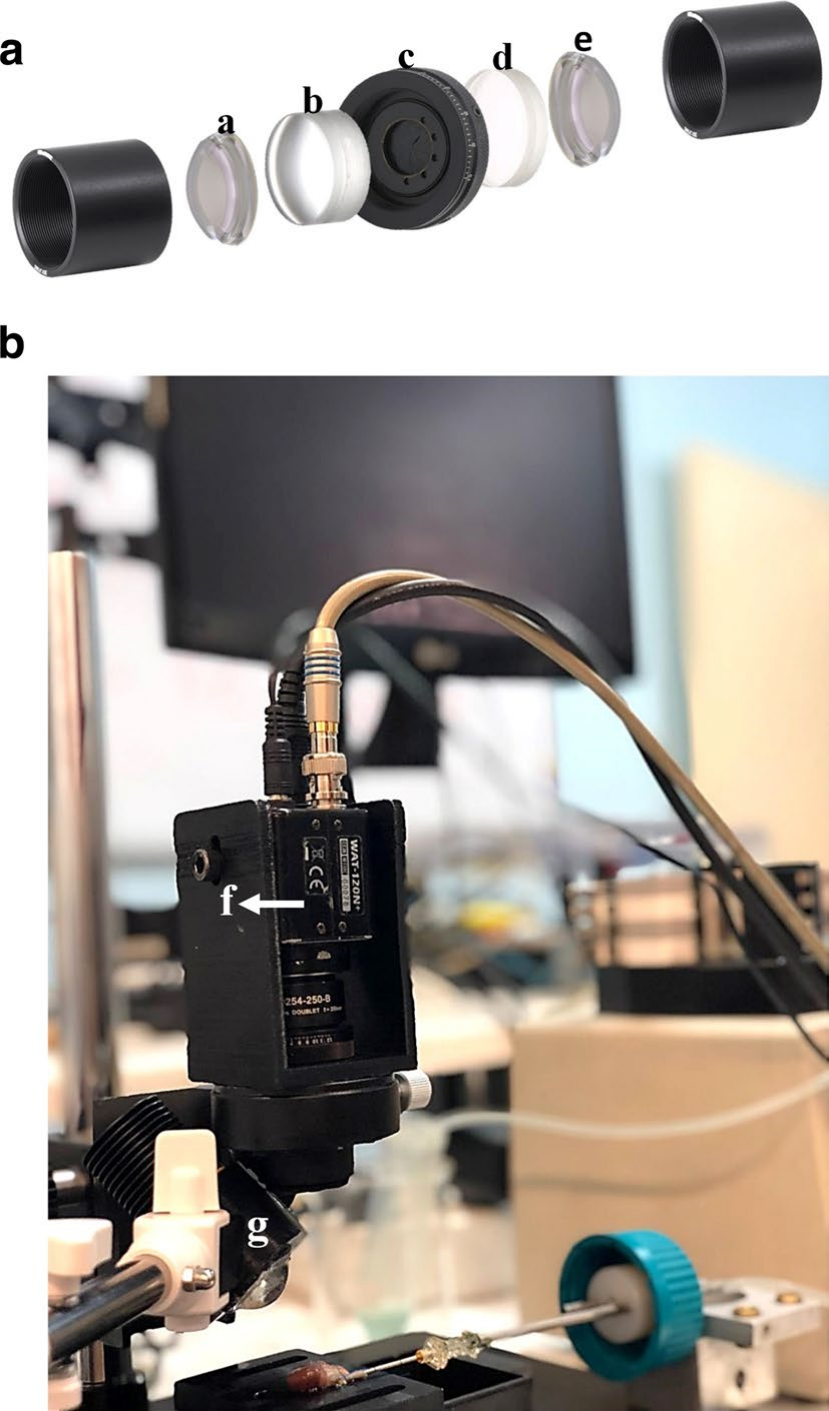
The design of fluorescent cardiac imaging (FCI) system. **a** Optical structure of the customized FCI system. a: meniscus lens with 100 mm focal length, b: achromatic doublet lens with 45 mm focal length c: diaphragm aperture d: achromatic doublet lens with 60 mm focal length e: meniscus lens with 100 mm focal length. **b** The image of final FCI device and its application during experiments. f: CCD camera, g: LED

The lenses, aperture, filters and CCD camera were housed in a custom-made 3D printed polydimethylsiloxane (PDMS) cage (Fig. 1a and b). The cage was vertically held by an articulating arm. The light-emitting diode (LED) was positioned by another articulating arm with a specific angle toward the specimen.

### Excitation/emission array

For near infrared (NIR) fluorescence excitation over the specimen, we used a mounted LED (M780L3, THORLABS, Newton, NJ) with specifications of IR color, peak wavelength at 780 nm with full-width at half-maximum (FWHM) of 28 nm and LED Output Power of 300 mW. LED was chosen over NIR lasers for two reasons of safety and cost. Based on our design, a light source with 4 mW/cm^2^ over a 2 cm diameter field of view (FOV), was required which necessitated higher safety regulations for personnel such as interlocked power. It is noteworthy that (1) the cost of a particular laser with such specifications would be much higher compared to a mounted LED; and (2) it does not generate a diffuse illumination over the specimen as LED does. We optimized the angle of incidence using Fresnel equations to reduce surface reflection and maximize transmission of light to enhance efficiency. Furthermore, we used an 18.4 mm aspherical lens (354280C, THORLABS, Newton, NJ) with anti-reflection coating (ARC) for the 650 nm to 1050 nm range, to provide a uniform illumination (Fig. 1b). In order to achieve the highest resolution and the least aberration, the minimum number of lenses was used. Two lenses (LE1234, THORLABS, Newton, NJ) were meniscus with 100 mm focal length and the other two were achromatic doublet (AC254-045-B/AC254-060-B, THORLABS, Newton, NJ), one with 45 mm and the other with 60 mm focal length. All four lenses had ARC for the 650 nm to 1050 nm wavelength range, thus they had 98% NIR transmission. A band-pass filter (FF01-832137-25, Semrock, USA) was used to block the excitation light and transmit the emission wavelength of ICG, with central wavelength of 832 nm and FWHM of 45.4 nm. We used an NIR fluorescence CCD camera (Watec, WAT-120N + (CCIR)) with adequate quantum efficiency at 800 nm (~ 30%) and dynamic range higher than 52 dB at 795 × 596 resolution, which was positioned over the heart in PDMS cage at a distance of 110 mm (Fig. 1b). However, the distance was calibrated for each measurement. Despite moderate quantum efficiency, this camera could achieve considerable performance.

### Modulation transfer function (MTF)

MTF was assessed using USAF 1951 (R1DS1P, THORLABS, Newton, NJ). Briefly, USAF1951 resolution target was imaged at the working distance of FCI system. The image was analyzed using a custom-made MATLAB software (R2016a, v9.0, https://www.mathworks.com/products/matlab.html) macro to identify MTF. MTF was then plotted against spatial frequency.

### Data processing

Online recording of FCI sequences was performed for 60 s at a rate of 25 frames per second and real-timely digitized using honestech TVR 2 (Honestech, US, https://honestech-tvr.apponic.com/). Furthermore, an image was captured at the end of ICG perfusion for further assessments. The image was pre-processed using ImageJ (Java 1.8.0_112, https://imagej.nih.gov/ij/index.html) for contrast enhancement.

**Fig. 2:**
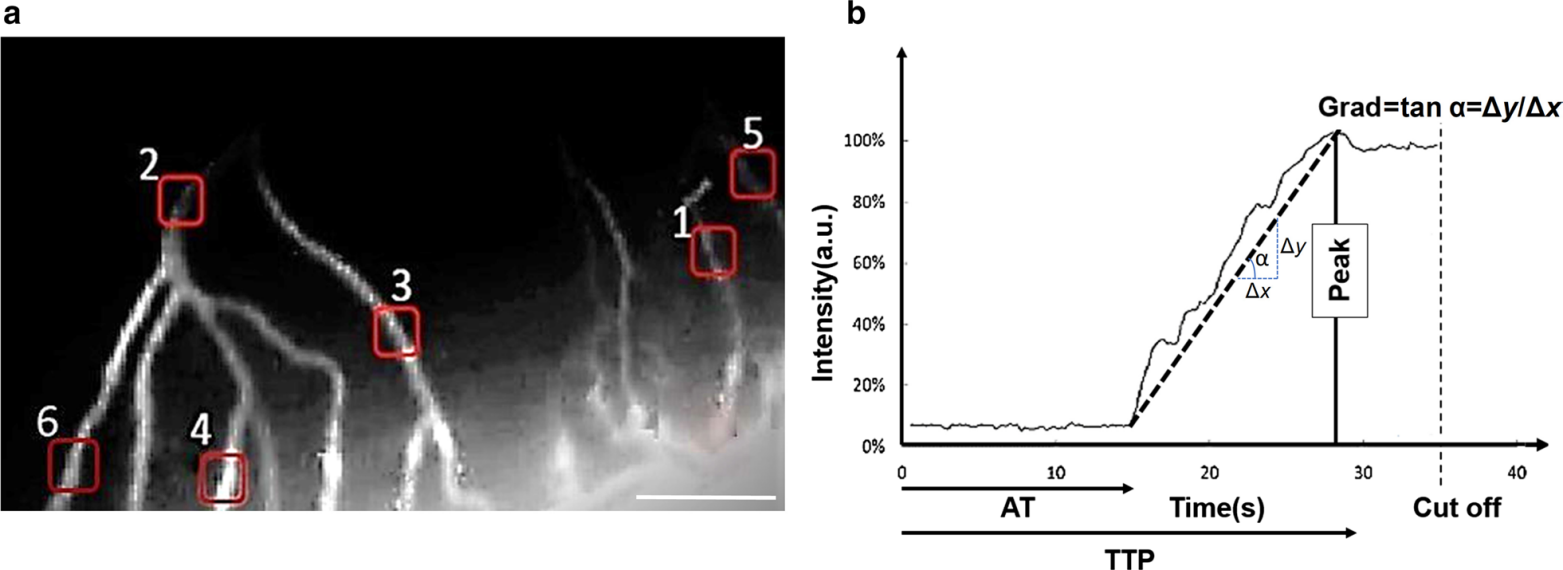
Quantitation of video angiography. **a** Six ROIs which were randomly selected at various levels of coronary circulation. Scale bar: 1 mm. **b** Time-dependent fluorescence intensity curve (TIC) showing intensity dynamics over the time. TIC was used to extract parameters such as peak intensity (PI), arrival time (AT), time to peak (TTP), blood volume (BV) and gradient (G) which represent the perfusion status

For quantitative assessment of ICG video angiography, background-subtracted peak fluorescence intensity (BSFI) was calculated using MATLAB software as previously described [8]. Briefly, the background intensity was subtracted from the peak fluorescence intensity during the first flow of the dye through the vessel for a region of 10*10 pixels as the region of interest (ROI) (Fig. 2a) and all calculated BSFIs for consecutive frames were plotted against time to produce time-dependent fluorescence intensity curve (TIC). TIC was used to extract parameters such as peak intensity (PI), arrival time (AT), time to peak (TTP), blood volume (BV) and gradient (G) which represent the perfusion status. Peak intensity was considered the maximum fluorescence intensity during ICG video angiography. Arrival time was the duration between ICG perfusion and ICG intensity at 5% of PI. Time to peak was the duration between ICG 5% and PI, and BV was calculated by integration of intensity between AT and the endpoint of first flow. G was the positive slope of TIC curve (Fig. 2b).

### Fluorescence angiography of acellular rat heart

Adult male *Wistar* rats (200–250 g) were used in this study. All animal studies were performed in accordance with guidelines approved by the Ethics Committee of Royan Institute in conformity with the NIH Guide for the Care and Use of Laboratory Animals. Rats were anesthetized by intraperitoneal injection of 100 mg/kg ketamine and 10 mg/kg xylazine. After systemic heparinization through the tail vein, the ascending aorta was dissected and cannulated with a blunted 20-gauge needle to allow retrograde coronary perfusion. The cannulated heart was kept at − 80 °C for 16 h to facilitate cell lysis. Before decellularization, frozen hearts were thawed at room temperature for 30 min and connected to a perfusion peristaltic pump (Rainin, Dynamax). Decellularization was carried out according to Lu et al. with some modifications [12]. Briefly, hearts were perfused with deionized water for 10 min at 1.5 ml/min; then, perfused twice with phosphate-buffered saline (PBS) (25 min each time), 0.02% trypsin/0.05% ethylenediaminetetraacetic acid (EDTA, Gibco) solution for 30 min, 1% sodium dodecyl sulfate (SDS, Sigma) solution for 10 h, and 3% Triton X-100 solution (Sigma) for 10 min, followed by a perfusion with PBS at room temperature. Disinfection was carried out by perfusion of 0.1% peracetic acid (Merck) and 4% ethanol for 10 min. The acid was neutralized and samples were rinsed using PBS (pH 7.4) and deionized water two times overnight. Decellularized hearts were immediately used or stored in PBS at 4 °C for future applications. ICG (VERDYE, Diagnostic Green, Germany) was prepared at the concentration of 0.2 mM and perfused retrogradely through an aortic cannula attached to a peristaltic pump (Rainin, Dynamax) with corresponding circuitry, for 1 min at 1.5 ml/min. Video angiography was performed using FCI system. Fluorescent angiography of ex vivo rat heart was performed using the same procedure as explained above, without acellularization. Briefly, after anesthesia induction and thoracotomy, the ascending aorta was dissected and cannulated, and perfused retrogradely by PBS for initial washing of the coronary arteries, followed by ICG perfusion and video angiography by FCI system. The coronary artery occlusion was induced by stopping peristaltic pump for a few seconds and restarting it. As a result, an air bubble entered the circuit and subsequently the coronary circulation. Thus, ICG perfusion was blocked from the point of air bubble occlusion.

## Results

### Aspherical lens uniformed the incident light

**Fig. 3:**
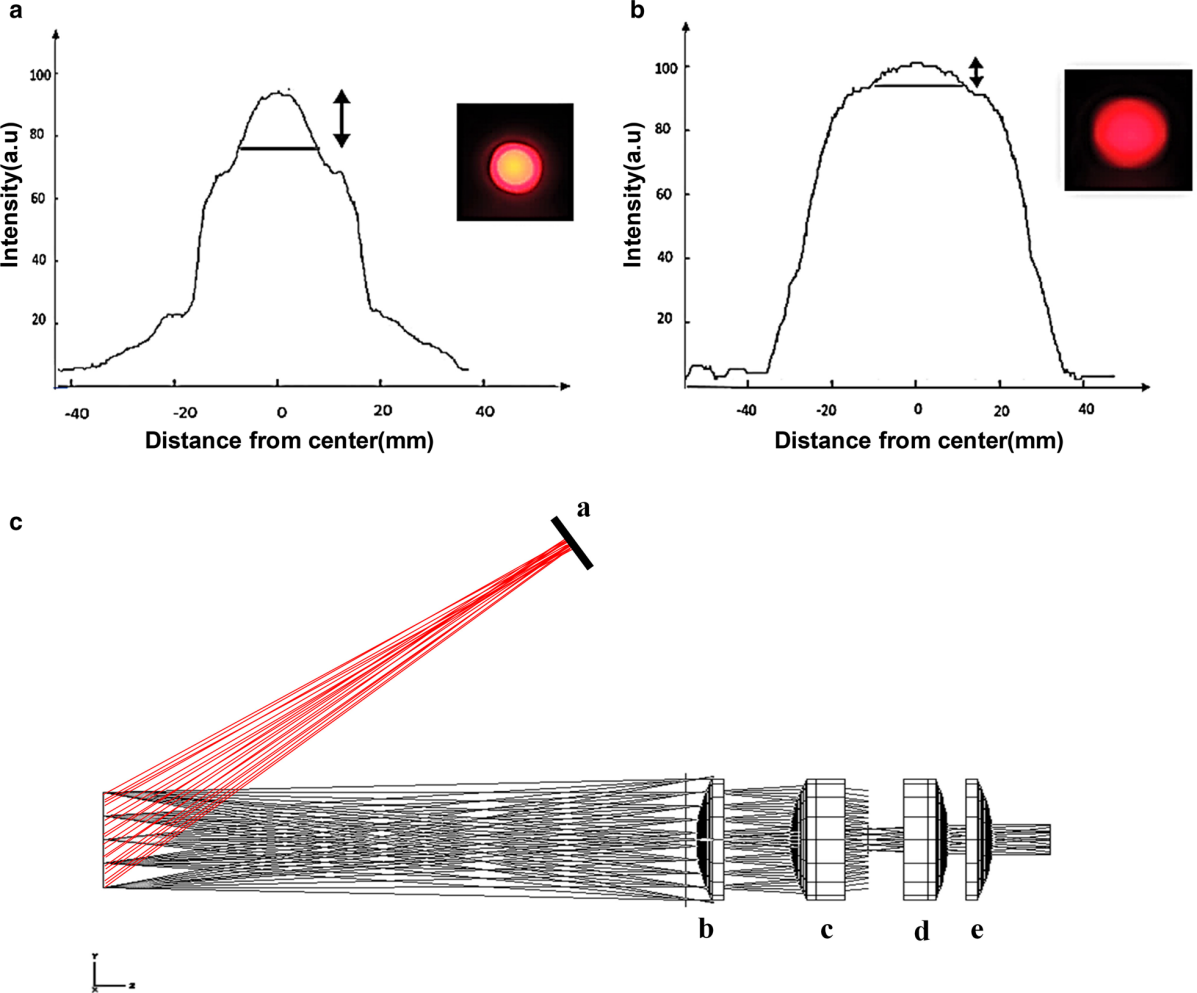
Optimization of optical setup. **a** Non uniform and **b** uniform light which emitted with and without aspherical lens in front of LED light. **c** Final design of optical components that were achieved by Zemax software. a: LED, b: meniscus lens with 100 mm focal length, c: achromatic doublet lens with 45 mm focal length d: achromatic doublet lens with 60 mm focal length e: meniscus lens with 100 mm focal length

In our in-house built FCI system, we used an aspherical lens in order to achieve uniform illumination. To evaluate the efficacy of the lens in uniform lightening, Images were captured at both aspherical lens inclusion and off-lens states. Figure 3a and b display representative images at lens-on and lens-off states where application of aspherical lens resulted in a homogenous light all over FOV. The quantification showed a substantial reduction in light intensity profile changes, where lens-off resulted in 20% intensity differences while lens-on led to a 4% change. Thus, employing aspherical lens improved light uniformity by fivefold.

Inspired by Zeiss Planar lens, two positive meniscus lenses were setup to minimize spherical aberration. Furthermore, two achromatic doublet lenses were placed between the meniscus lenses to eliminate chromatic aberration. In addition, the distance between lenses and the apparatus diameter was determined using Zemax software (OpticStudio 18.4.1, https://my.zemax.com) (Fig. 3c).

**FCI system was optimized for rat coronary angiography by MTF analysis**

**Fig. 4:**
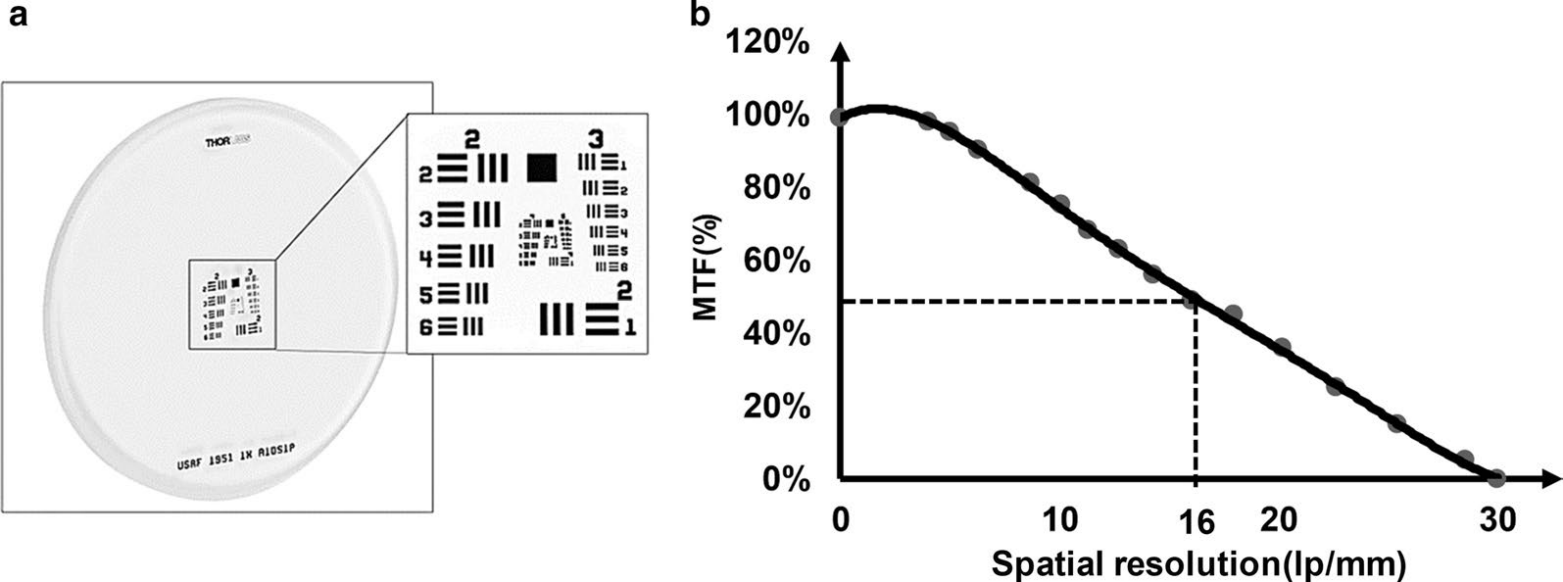
Evaluation of MTF. **a** USAF 1951 target used for plotting MTF and **b** MTF of fluorescent cardiac imaging system. The 50% MTF frequency (ƒ_50_) is about 16 lp/mm

To evaluate the resolution of optical system for ICG video angiography of rat hearts with low-diameter microvasculature, MTF of FCI was plotted using USAF 1951 (Fig. 4a). As depicted in Fig. 4b, cut-off frequency was achieved at 30 line pairs per mm (lp/mm). Maximum MTF was obtained at the image plane that corresponded to a sharp image quality and a high spatial resolution of 16 lp/mm (62.5 µm). This resolution is sufficient for video angiography of rat hearts where mean diameter of coronary arteries at the septal branch of left anterior descending coronary artery (LAD), is around 100 µm and the minimum identified diameter of coronary arteries was reported to be about 50 µm [13, 14].

**FCI system could successfully visualize microvasculature and coronary flow**

**Fig. 5:**
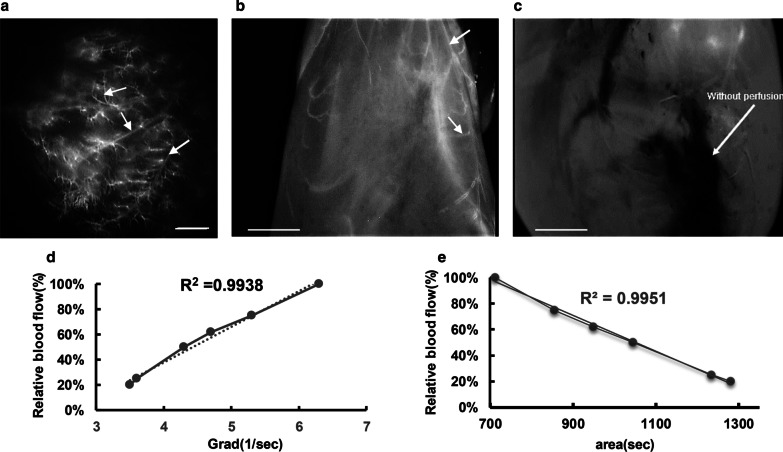
Application of FCI system for video angiography in ex vivo rat hearts. **a** Image of LAD and septal branches captured by FCI system. **b** Image of ICG perfusion in myocardium. White arrows show blood vessels. **c** FCI system identified the non-perfused region where the ICG flow was blocked. Scale bar: 2 mm. **d** and **e** The correlation plots for relative blood flow with either gradient or blood volume. The experiment was repeated 4 times for acellular and 3 times for whole rat heart

Ex vivo rat hearts were subjected to video imaging by using our FCI system. Prior to ICG perfusion, a background image was captured. With the onset of ICG perfusion, video angiography was started and continued until full perfusion of coronary circulation (Additional file 2: Supplementary movie 1). FCI system could visualize LAD and septal branches as well as small coronary arteries (Fig. 5a). Furthermore, in the instance of coronary occlusion in one ex vivo heart, ICG was perfused in myocardium (Fig. 5b) and video angiography with FCI system identified the non-perfused region where the flow was blocked (Fig. 5c). Relative coronary arteries’ flow was indirectly identified by assessing luminance intensity through the vessel. Furthermore, six ROIs were randomly selected at various levels of coronary circulation (Fig. 2a) and their corresponding TICs were plotted (Additional file 1: Fig S1). Table 1 represents the parameters calculated using TIC and their correlation with relative blood flow in which showed an > 99% correlation with both gradient and blood volume (Fig. 5d and e), confirming the applicability of FCI system for preclinical animal angiography studies.

**Table 1:** Arrival time (AT), time to peak (TTP), gradient (G) and blood volume (BV) calculated from TICs of six ROIs

Parameters	AT (s)	TTP (s)	Grad (${\mathrm{s}}^{-1}$)	BV (s)
ROI 1	4.3	32.5	3.4	1282
ROI 2	20.4	35.5	6.3	712
ROI 3	6.6	26.7	4.7	950
ROI 4	8.8	30.9	4.3	1045
ROI 5	7.3	32.7	3.7	1235
ROI 6	20.4	37.8	5.4	855

## Discussion

Cardiovascular diseases (CVD) impose tremendous burden on health system due to the vital role of the cardiovascular system in terms of blood pumping and circulation [15]. Animal models have greatly enhanced our understanding of CVD mechanisms and helped in development of related therapies [16]. Accordingly, establishment of disease models and corresponding assessments require development of efficient preclinical tools and devices. Furthermore, most of new drugs, therapies and devices are examined before any clinical applications [17]. Intraoperative angiography has been always a desired tool for surgeries dealing with vascular grafts specifically coronary bypass grafts, due to complications of these surgeries, where it could greatly help in more successful operations and avoiding re-operations [18]. In this regard, optical imaging based on fluorescent dyes has been also examined for its efficacy and applicability. We designed an FCI system for preclinical intraoperative angiography in rodents. This system used LED as the excitation light source in contrast to previously applied high laser powers. Furthermore, it could provide high-resolution angiography of coronary arteries in small animal models by employing an appropriate optical design which benefitted from a collimation lens for homogenizing the excitation light.

Excitation of ICG by monochromatic LED could solve the unnecessary application of the wide light spectra, because it specifically provides the corresponding excitation spectrum for a particular fluorescent molecule [19]. Thus, light emitting diode could play a significant role in development of novel biomedical optical devices, since it avoids multiple excitation filters used for blocking unnecessary spectra and possesses high luminous efficacy, lower cost and energy expenditure [20]. PDE-NEO II represents one of such devices where LED is employed for intraoperative angiography and could satisfy the expectations of clinic in several applications including lymphography [21], hepatoblastoma [22] and supraclavicular flaps [23]. Yamamoto et al. also used a HyperEye medical system (HEMS) based on NIR LED light for assessment of CABG procedure [24]. In addition to inclusion of NIR LED, we designed 45-degree angle for light source to minimize the reflection from target tissue and maximize the transmitted light, based on Fresnel equations.

ICG angiography system has been regarded as a new successful era in vascular imaging, however suffered from limitations of determining the degree of occlusion in the instances of thrombosis or degree of stenosis in CABG [6, 25]. Detter et al. surveyed the quantitation of ICG video angiography and determined two key parameters namely, background-subtracted peak fluorescence intensity (BSFI) and slope of fluorescence intensity (SFI). BSFI and SFI could very well determine myocardial perfusion, a parameter appeared to correlate well with the severity of coronary artery stenosis [8]. In continuum, they introduced quantitative intraoperative fluorescence intensity (QIFI) as a function of time-intensity curves (TICs) which could indirectly determine the quality of bypass graft with respect to all degrees of bypass stenoses [26]. Moreover, Yamamoto et al. employed the TICs rise and average acceleration values to evaluate the blood flow and thus graft patency as an output of HEMS, however lower grades of stenosis could not be determined [11]. We also measured the luminance intensity through coronary artery as a representative of blood flow. Initially, we calculated the area under TIC curve which could represent blood volume as described by Kamada et al., as well as gradient of TIC curve from 5% of ICG intensity to peak intensity [27, 28]. Blood flow is a function of vascular resistance where the resistance is inversely proportional to vessel’s radiuses [29]. Therefore, we used the relative vessel radius obtained by assessing fluorescence intensity, as a representative of blood flow and plotted it against blood volume and gradient to assess the degree of correlation. As our findings indicated > 99% correlation, TIC-calculated parameters can be used to quantitatively evaluate the blood flow. Although we could not establish a graded occlusion model in ex vivo rat heart, our results might suggest a novel approach for evaluation of the bypass grafts’ patency.

## Conclusion

We designed a preclinical ICG angiography system which might facilitate establishment and evaluations of animal models of cardiovascular diseases such as myocardial infarction and coronary artery grafts. Intraoperative ICG angiography could be also used for animal models dealing with vascular complications.

## Supplementary Information

**Additional file 1.** Image of FCI device. 1: LED, 2: Specimen stage corresponding to surgical field, 3: Band-pass filter, 4: Optical structure, 5: CCD camera 6: Cage holding FCI system.

**Additional file 2.** Time-dependent fluorescence intensity curves (TICs) for 6 ROIs which were selected randomly at various levels of coronary circulation..

**Additional file 3.** Video angiography in *ex vivo* rat heart using FCI system.

**Additional file 4.** Distances between optical components that were achieved by Zemax software.

## Data Availability

All data generated or analyzed during this study are included in this published article [and its supplementary information file].
