# Supplementary material for: A novel fluorescent cardiac imaging system for preclinical intraoperative angiography
Source: BMC Med Imaging. 2021 Feb 25;21:37. doi: 10.1186/s12880-021-00562-y (PMC7905866; doi:10.1186/s12880-021-00562-y)
Supplement: Supplementary file 1 — Additional file 1. Image of FCI device. 1: LED, 2: Specimen stage corresponding to surgical field, 3: Band-pass filter, 4: Optical structure, 5: CCD camera 6: Cage holding FCI system. [file 12880_2021_562_MOESM1_ESM.docx]

**A Novel Fluorescent Cardiac Imaging System for Preclinical Intraoperative Angiography**

Sara Mashalchi^1^, Sara Pahlavan^2*^, Marjaneh Hejazi^1,3*^

^1^ Medical Physics and Biomedical Engineering Department, School of Medicine, Tehran University of Medical Sciences, Tehran, Iran

^2^ Department of Stem Cells and Developmental Biology, Cell Science Research Center, Royan Institute for Stem Cell Biology and Technology, ACECR, Tehran, Iran

^3^ Research Center for Molecular and Cellular Imaging, Bio-optical Imaging Group, Tehran University of Medical Sciences, Tehran, Iran

*Corresponding Authors:

Sara Pahlavan, Royan Institute, Banihashem Sq., Banihashem St., Resalat Highway, P.O. Box: 16635-148, Tehran, Iran. Postal Code: 1665659911, Tel: +98 21 23562504, Fax: +98 21 23562507, Email: [sarapahlavan@royaninstitute.org](mailto:sarapahlavan@royaninstitute.org)

ORCID: [0000-0002-8854-2626](https://orcid.org/0000-0002-8854-2626)

Marjaneh Hejazi, Medical Physics and Biomedical Engineering Department, School of Medicine, Tehran University of Medical Sciences, Tehran 1417613151, Iran, [mhejazi@sina.tums.ac.ir](mailto:mhejazi@sina.tums.ac.ir)

Supplementary Material

# Supplementary Figures and Movies

## Supplementary Figures


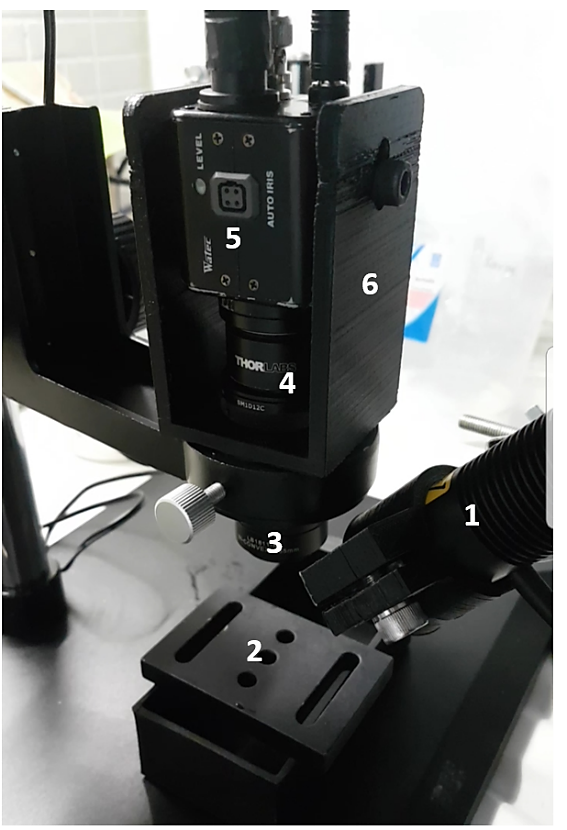


**Figure S1.** Image of FCI device. 1: LED, 2: Specimen stage corresponding to surgical field, 3: Band-pass filter, 4: Optical structure, 5: CCD camera 6: Cage holding FCI system.


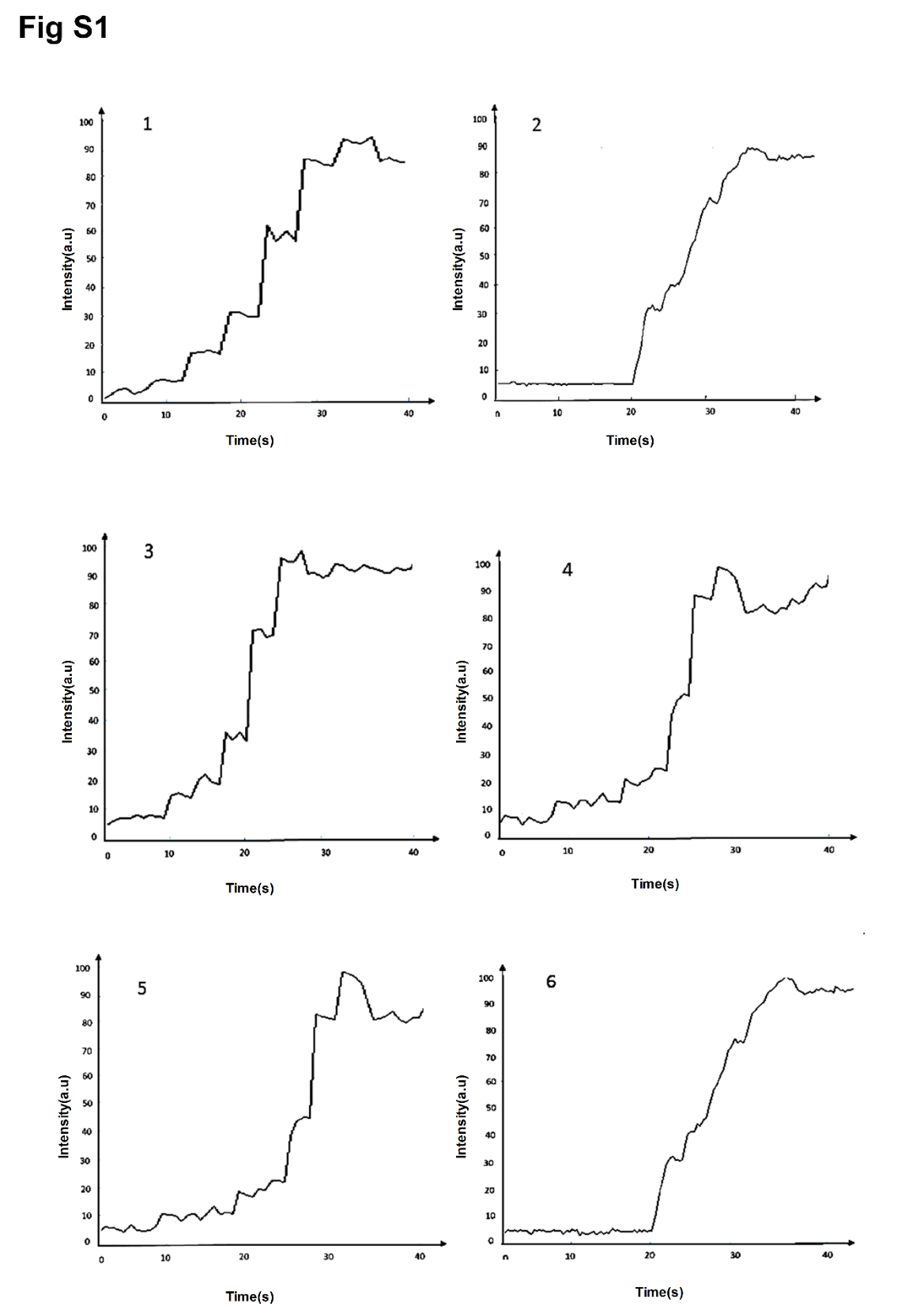


**Figure S2.** Time-dependent fluorescence intensity curves (TICs) for 6 ROIs which were selected randomly at various levels of coronary circulation.

## Supplementary Movie

**Movie S1.** Video angiography in *ex vivo* rat heart using FCI system

## Supplementary Table

Table S1. Distances between optical components that were achieved by Zemax software.

| **Distance (mm)** | **Optical Components** |  |
| --- | --- | --- |
| 12.5 | Meniscus lens (f=100 mm) and achromatic doublet lens (f= 45mm) | **1** |
| 10 | Achromatic doublet lens (f= 45mm) and achromatic doublet lens (f=60 mm) | **2** |
| 4 | Achromatic doublet lens (f=60 mm) and meniscus lens (f=100 mm) | **3** |
| 13.5 | Meniscus lens (f=100 mm) and CCD camera | **4** |
