# Supplementary figures and images for: A novel fluorescent cardiac imaging system for preclinical intraoperative angiography
Source: BMC Med Imaging. 2021 Feb 25;21:37. doi: 10.1186/s12880-021-00562-y (PMC7905866; doi:10.1186/s12880-021-00562-y)

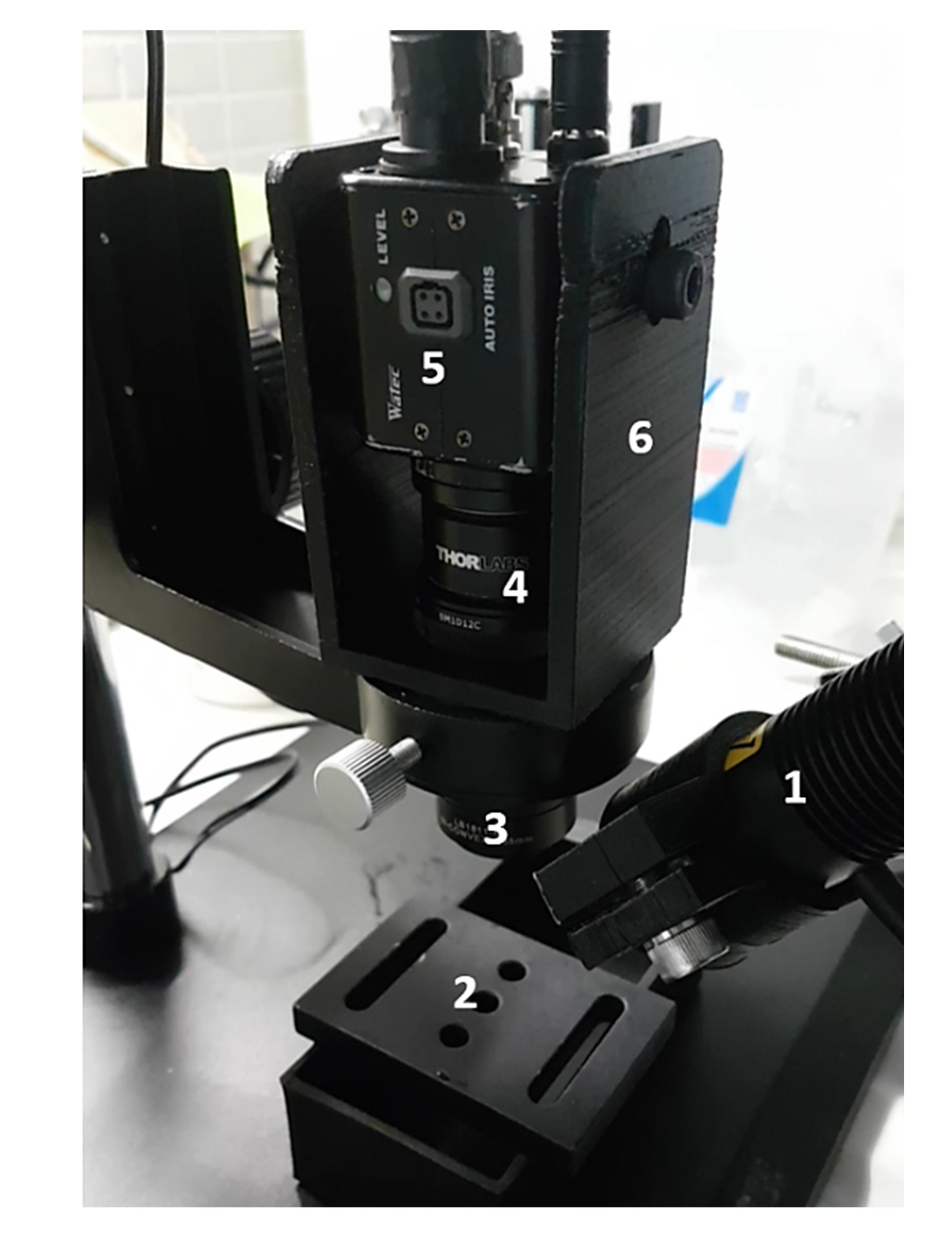

Supplement: Supplementary file 3 — Additional file 3. Video angiography in ex vivo rat heart using FCI system. [file 12880_2021_562_MOESM3_ESM.jpg]

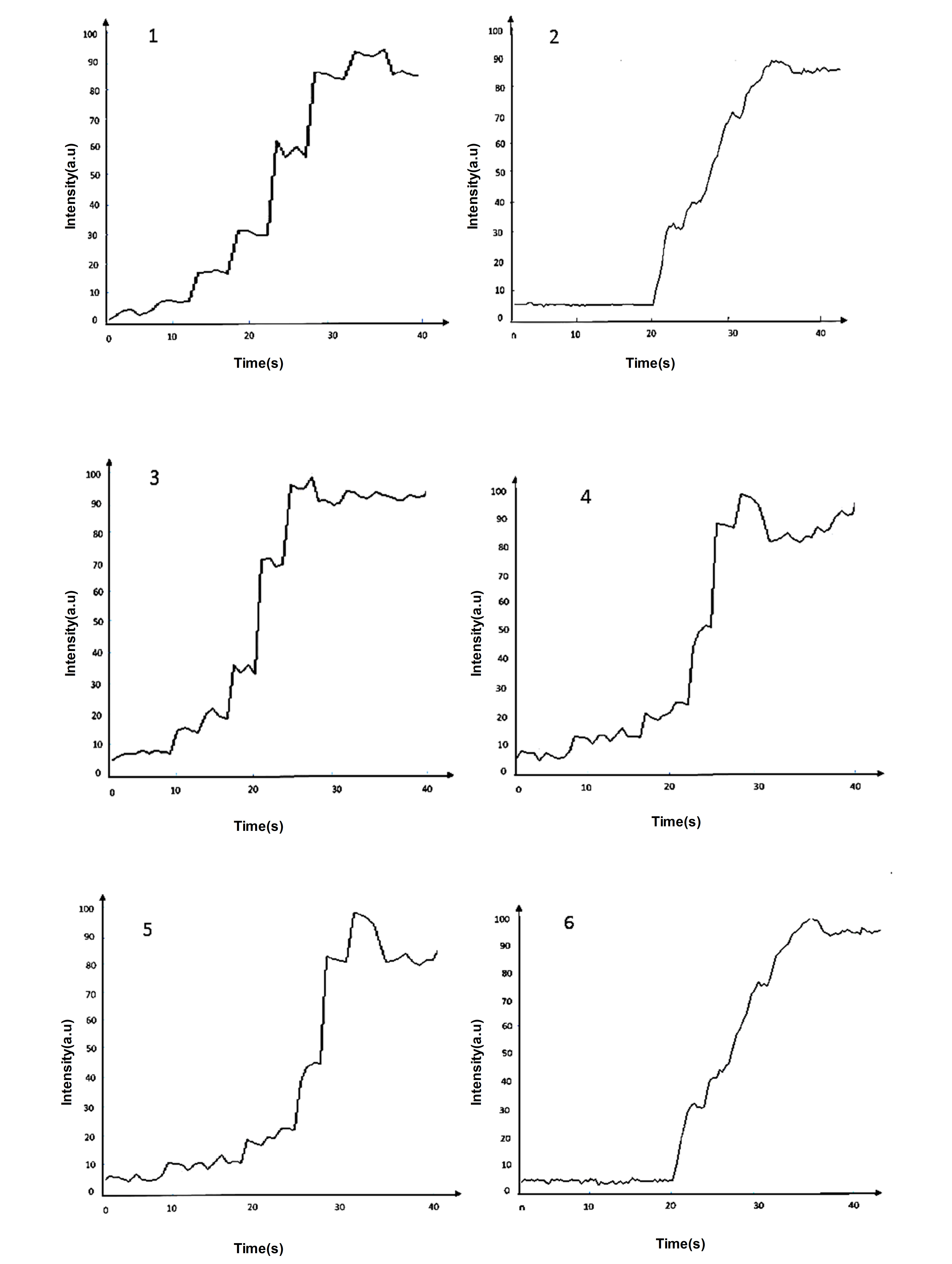

Supplement: Supplementary file 4 — Additional file 4. Distances between optical components that were achieved by Zemax software. [file 12880_2021_562_MOESM4_ESM.jpg]
